# The Marriage Law and Mental Disease

**Published:** 1914-04-04

**Authors:** 


### THE MARRIAGE LAW AND MENTAL DISEASE.

The law does not sanction dissolution of marriage on the ground of insanity supervening after the marriage is contracted, whatever the consequences may be to the parties or to the race. If the spouses were of sound contracting mind at the date of the marriage, then they are married for better and for worse. But the very essence of the contract of marriage is consent, and there can be no consent binding in law without soundness of mind. Yet when a marriage has been regularly solemnised, there is a strong presumption in favour of the parties having been fully capable, and if after such solemnisation one party seeks dissolution on the ground of the mental incapacity of the other, the onus on him or her is a very heavy one. The extraordinary case of Park *v.* Park, 1914, 1 S.L.T. 89, however, is an instance of that onus being successfully discharged. The ceremony of marriage took place on January 3, 1911; on January 14, 1911, the wife was found to be insane, and confined in an asylum, where she remained till November 30, 1912, when she was discharged as "recovered." In 1913 the husband raised action to have it declared that at the date of the marriage the wife was of unsound mind, and incapable of appreciating the nature and obligation of the contract of marriage. The Court (Lord Dewar, Ordinary) have found in his favour and dissolved the marriage, or rather found that it was *ab initio* null and void.

The medical evidence and the circumstances of the wife's behaviour before and after marriage are minutely reviewed in the Judge's opinion, and he comes to the finding mentioned; and that, too, notwithstanding the fact that the wife defended the case, and denied the pursuer's statements. The Lord Ordinary, however, dealt with her evidence as follows, and we infer that the fact that she had been insane detracted from the value of her testimony: "The pursuer, she said, was always good to her, and she cannot believe it possible now that she is well that she showed aversion to him; it seems so unreasonable and unnatural and contrary to what she now feels. So it was, and that is why I think it was due to unsoundness of mind. It sprang from a delusion, and it was, in my opinion, a delusion which rendered her incapable of appreciating the nature of the contract of marriage and the duties and responsibilities which it creates. There is nothing in the case to explain the fact that she refused from the very beginning to fulfil these duties and responsibilities except this delusion, and I am accordingly of opinion that the pursuer is entitled to decree."

Liability in damages may arise either in consequence of breach of contract, or from breach of duty, and it is sometimes difficult to distinguish when it is due to the one and when to the other. In well-known leading cases in England and Scotland it has been held that the wife of a tenant cannot maintain an action against a landlord for his failure to perform the duty of providing a habitable house, as under the lease he had contracted to do. She was no party to that contract, and therefore could not sue for breach of it. Now the same principle was urged in defence in Edgar *v.* Lamont, 1914, 1 S.L.T. 80, in which a married woman sued a doctor for damages for personal injury due to alleged negligent and incompetent treatment. The doctor had been employed by pursuer's husband, there thus being no contract between pursuer and defender. The Court, however, upheld the pursuer's title to sue, and sent the case to jury trial. Lord Salvesen, in giving the judgment of the Court, said: "There is a series of decisions in England to establish the proposition that a patient is entitled to a direct action *ex delicto* against a doctor for professional incompetence or negligence, and that no action lies in respect of the injuries so sustained at the instance of the person (other than the patient) who contracted for the doctor's services. I think that view is consistent with our own law and with common sense. To hold otherwise would lead to an entire denial of any remedy to a person who did not happen to be the person who had contracted with the doctor, and I should be very slow indeed to arrive at that result."

Chisholm *v.* Grant, 1914, 1 S.L.T. 63, was a slander action in which Chisholm, the superintendent of the Banff District Asylum, claimed damages against Grant, the town clerk of Banff, averring that at a local railway station Grant had said, "What does that maniac Chisholm know about treating lunatics? He is just a quack. We will sack him yet," which statement pursuer innuendoed as meaning to convey that the pursuer was unfit for his duties as asylum superintendent, was not properly qualified for his work, and ought to be dismissed. The defender argued that the word "quack" meant merely that pursuer was not a duly qualified medical man (which admittedly was the case), and in that sense the term was not slanderous. The Court quite allowed that that was one meaning of the term "quack," but it had more meanings than one, and the sense in which the pursuer averred it was used was another possible meaning. The question in which sense it was used was left to the jury, and the case was sent to trial.

